# Evolution of Choroidal Neovascularization due to Presumed Ocular Histoplasmosis Syndrome on Multimodal Imaging including Optical Coherence Tomography Angiography

**DOI:** 10.1155/2018/4098419

**Published:** 2018-02-13

**Authors:** T. Y. Alvin Liu, Alice Yang Zhang, Adam Wenick

**Affiliations:** ^1^Retina Division, Wilmer Eye Institute, Johns Hopkins University, Baltimore, MD, USA; ^2^Department of Ophthalmology, University of North Carolina at Chapel Hill, Chapel Hill, NC, USA

## Abstract

A 37-year-old Caucasian woman presented with acute decrease in central vision in her right eye and was found to have subfoveal choroidal neovascularization (CNV) due to presumed ocular histoplasmosis syndrome (POHS). Her visual acuity improved from 20/70 to 20/20 at her 6-month follow-up, after 3 consecutive monthly intravitreal bevacizumab injections were initiated at her first visit. Although no CNV activity was seen on fluorescein angiography (FA) or spectral-domain optical coherence tomography (SD-OCT) at her 2-month, 4-month, and 6-month follow-up visits, persistent flow in the CNV lesion was detected on optical coherence tomography angiography (OCTA). OCTA shows persistent vascular flow as well as changes in vascular flow in CNV lesions associated with POHS, indicating the continued presence of patent vessels and changes in these CNV lesions, even when traditional imaging of the lesion with OCT and FA indicates stability of the lesion with no disease activity. Additional cases with longitudinal follow-up are needed to assess how OCTA should be incorporated into clinical practice.

## 1. Introduction

Presumed ocular histoplasmosis syndrome (POHS) can lead to central vision loss with development of choroidal neovascularization (CNV). Herein, we report a case of POHS related CNV that was successfully treated with intravitreal bevacizumab. The evolution of the CNV lesion on multimodal imaging, including optical coherence tomography angiography (OCTA), will be presented.

## 2. Case Presentation

**Figure 1:**
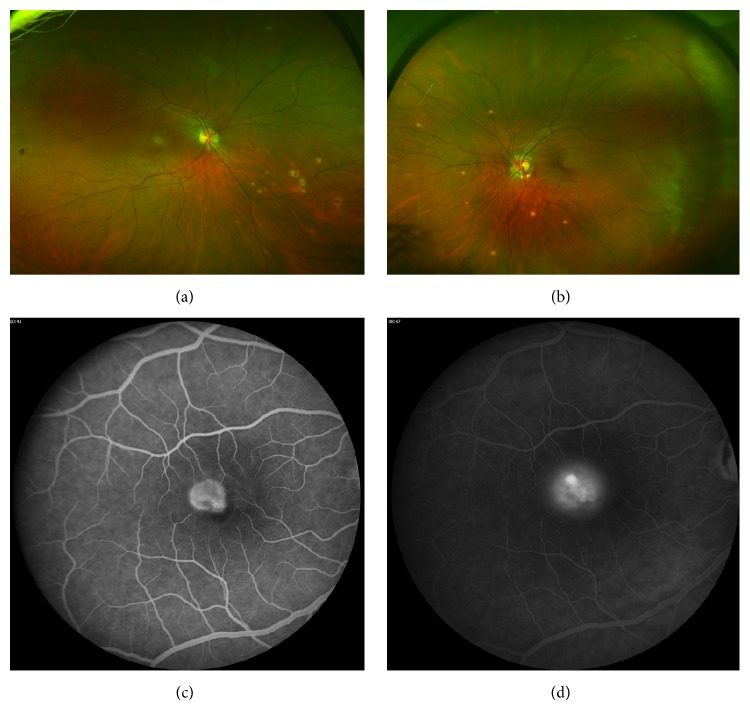
Ultra-wide-field color photographs of the right eye (a) and left eye (b) at presentation showing peripapillary areas of hyper- and hypopigmentation and punched-out chorioretinal lesions in the midperipheral retina, findings classic for presumed ocular histoplasmosis syndrome. 30-degree fluorescein angiography of the right eye showed abnormal early hyperfluorescence in the fovea in the early frame (c) and intense leakage in the late frame (d).

**Figure 2:**
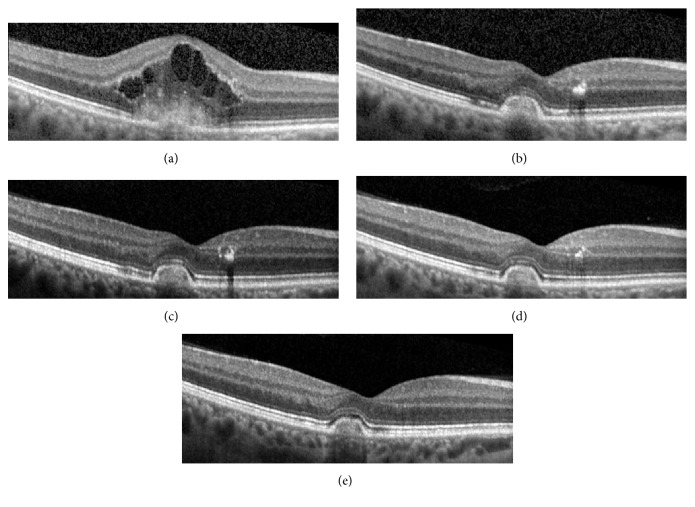
Spectral-domain optical coherence tomography images obtained at the patient's baseline visit (VA 20/70, CST 442, bevacizumab #1 given) (a); 1-month visit (VA 20/40, CST 263, bevacizumab #2 given) (b); 2-month visit (VA 20/32, CST 259, bevacizumab #3 given) (c); 4-month visit (VA 20/25, CST 253) (d); 6-month visit (VA 20/20, CST 257) (e). Between her 2-month and 6-month visits, the appearance of the fibrovascular PED remained stable with gradual decrease in the parafoveal intraretinal lipid. There was no recurrence of SRF or CME or change in PED size between her 4-month and 6-month visits, despite the lack of treatment. VA = visual acuity; CST = central subfield thickness; PED = pigment epithelial detachment; SRF = subretinal fluid; CME = cystoid macular edema.

**Figure 3:**
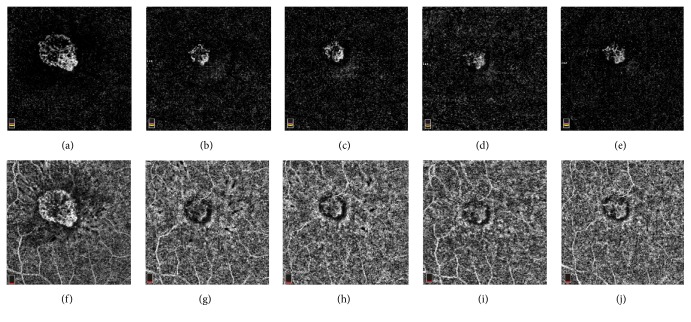
Optical coherence tomography angiography images obtained at the patient's baseline visit (VA 20/70, bevacizumab #1 given) (a, f); 1-month visit (VA 20/40, bevacizumab #2 given) (b, g); 2-month visit (VA 20/32, bevacizumab #3 given) (c, h); 4-month visit (VA 20/25) (d, i); 6-month visit (VA 20/20) (e, j). (a) to (e) represented the outer retinal layer, while (f) to (j) represented the choriocapillaris layer. At her 4-month visit, the superior aspect of the CNV lesion (d) continued to regress following her bevacizumab #3 treatment given 2 months prior, while there was no change in the corresponding FA and OCT images. At her 6-month visit, there was subtle expansion of the superior aspect of the CNV lesion (e), while the corresponding FA and OCT images remained stable.

A 37-year-old Caucasian woman, with no past medical or ocular history, presented with acute-onset and decreased central vision in the right eye. Her left eye was asymptomatic. Visual acuities of the right eye and left eye measured 20/70 and 20/20, respectively, with no improvement with pinhole. The anterior segment was normal with no intraocular inflammation in both eyes. Dilated fundus examination of both eyes showed peripapillary hyper- and hypopigmentation and punched-out chorioretinal lesions in the midperipheral retina consistent with a diagnosis of POHS (Figures 1(a) and 1(b)). Additionally, the right eye showed foveal retinal thickening with trace subretinal hemorrhage and parafoveal intraretinal lipid. Fluorescein angiography (FA) of the right eye showed early hyperfluorescence in the fovea with intense leakage in the late frames, consistent with the presence of CNV (Figures 1(c) and 1(d)). Spectral-domain optical coherence tomography (SD-OCT) of the right eye showed a foveal fibrovascular pigment epithelial detachment (PED) with overlying subretinal hyperreflective material and cystoid macular edema (CME). A diagnosis of choroidal neovascularization (CNV) in the right eye due to presumed ocular histoplasmosis syndrome (POHS) was made. Intravitreal bevacizumab therapy was initiated with close follow-up using serial SD-OCT and OCTA images, shown in Figures 2 and 3, respectively.

At her 1-month follow-up, the visual acuity of the right eye improved from 20/70 to 20/40, with resolution of subretinal hemorrhage and near resolution of CME. Intravitreal bevacizumab #2 was given.

**Figure 4:**
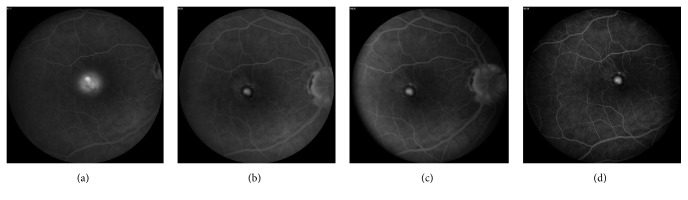
Fluorescein angiography (late frame) at the patient's baseline visit (VA 20/70, bevacizumab #1 given) (a); 2-month visit (VA 20/32, bevacizumab #3 given) (b); 4-month visit (VA 20/25) (c); 6-month visit (VA 20/20) (d). After the 2-month visit, there is complete resolution of leakage. VA = visual acuity.

At her 2-month follow-up, the visual acuity of the right eye improved to 20/32, with no subretinal fluid (SRF) or CME on SD-OCT and resolution of leakage on FA (Figure 4). However, optical coherence tomography angiography (OCTA) showed persistent flow within the CNV lesion. Hence, a decision was made to administer intravitreal bevacizumab #3.

At her 4-month follow-up, the visual acuity of the right eye improved to 20/25, with stable findings on SD-OCT and FA. Although there was persistent flow within the CNV lesion on OCTA, observation was opted given her excellent visual acuity.

At her 6-month follow-up, her right eye visual acuity further improved to 20/20, with no CNV activity seen on SD-OCT or FA. The area of vascular flow on OCTA appeared to have enlarged slightly, but cautious observation was opted for again given her excellent visual acuity.

## 3. Discussion

Our patient's fundus examination was consistent with classic POHS. Although she did not have a history of spelunking or living in the Mississippi/Ohio river valleys, she endorsed exposure to farm animals. The CNV in her right eye responded well to intravitreal bevacizumab treatment. Her visual acuity improving from Snellen 20/70 to 20/20 over a span of 6 months, with only 3 monthly injections initiated at her presentation. Our experience was consistent with that of published case series, which showed intravitreal antivascular endothelial growth factors (VEGF) injections to be effective for POHS related CNV; on average, patients' visual acuity improved requiring relatively few treatments (range 2.6 to 7 injections per patient per year) [1–4] as compared to neovascular age-related macular degeneration (AMD).

Previously, Wang et al. [5] reported that the peripheral punched-out chorioretinal lesions in POHS corresponded to areas of focal flow loss in the choriocapillaris and deeper choroidal layers on OCTA. To the authors' knowledge, our report is the first reported case on the OCTA evolution of CNV due to POHS. Using the classification developed by Coscas et al. [6] for neovascular AMD, our patient's CNV lesion will be classified as Pattern I based on the presence of (1) a well-defined lacy-wheel CNV lesion, (2) numerous branching tiny capillaries, (3) peripheral arcade and (4) perilesional hypointense halo at the level of the choriocapillaris.

The serial multimodal images obtained for our patient also provided several interesting observations. First, although no CNV activity was seen on FA or SD-OCT at her follow-up visits, persistent flow of the CNV lesion was detected on OCTA. This suggests that OCTA is more sensitive than FA or SD-OCT in detecting the presence of persistent CNV related to POHS. This observation parallels the work published by de Oliveira Dias et al. [7], in which subclinical macular neovascularization was detected by swept source OCTA in eyes with nonexudative AMD. Second, the CNV lesion in our patient decreased substantially in size between her first visit and 1-month follow-up, after the first bevacizumab treatment. However, the central portion of the CNV lesion remained essentially unchanged between her 1-month and 4-month follow-up visits, despite 2 additional anti-VEGF injections. This is consistent with the observation by Lumbroso et al. [8] that the central trunk of CNV, as compared to the smaller peripheral vessels, can be relatively resistant to anti-VEGF treatments. Third, it was interesting to note that while no recurrence of CNV activity was seen on FA or SD-OCT at our patient's 6-month visit, expansion of the area of flow in the CNV lesion was detected on OCTA. While this was not surprising, given treatment was deferred at the patient's 4-month visit, this suggests that OCTA is more sensitive than FA or OCT in detecting very early increase or recurrence of CNV activity. However, it also shows that an increase in CNV activity on OCTA does not necessarily translate into clinically significant or visually significant changes.

## 4. Conclusion

To the authors' knowledge, this is the first reported case of OCTA findings in CNV related to POHS. Our case suggests that OCTA could be useful in the management of POHS related CNV, similar to neovascular AMD, in that OCTA seems to be more sensitive than FA and SD-OCT in detecting subtle changes in CNV lesions. What remains unknown is whether the treatment based on subtle OCTA findings, in the absence of fluid on SD-OCT, ultimately translates to better outcomes. Additional cases of CNV related to POHS with longitudinal follow-up imaged with OCTA are needed to assess how OCTA should be incorporated into clinical management decisions.
